# Treatment of unruptured middle cerebral artery aneurysms: Systematic review in an attempt to perform a network meta-analysis

**DOI:** 10.3389/fsurg.2022.1005602

**Published:** 2022-09-28

**Authors:** Ignacio Arrese, Sergio García-García, Santiago Cepeda, Rosario Sarabia

**Affiliations:** Neurosurgery Department, Hospital Universitario Río Hortega, Valladolid, Spain

**Keywords:** unruptured, aneurysm, endovascular, surgery, systematic review, network meta-analysis

## Abstract

**Objective:**

Open surgical clipping has been generally considered the best treatment option for unruptured middle cerebral artery aneurysms (uMCAAs). However, this type of aneurysm is being treated endovascularly with the appearance of new devices. We have carried out a systematic review of randomized and quasi-experimental studies to conduct a network meta-analysis (NMA) to assess the safety and efficacy of the different treatment methods currently used in uMCAAs.

**Methods:**

The literature was searched by using PubMed and Google Scholar databases. Eligibility criteria were randomized or quasi-experimental studies including at least five cases per arm and reporting duration of follow-up and number of lost cases. The end points were: angiographic success, final neurological outcome, and the need for retreatments.

**Results:**

We could only analyze four quasi-experimental studies with 398 uMCAAs. All of them compared clipping vs. coiling. Clipping showed better results than coiling in all analyzed end points. We could not conduct the proposed NMA because of the absence of randomized or quasi-experimental studies. Instead, a systematic review is further discussed.

**Conclusions:**

There is an urgent need for comparative studies on the treatment of uMCAAs.

## Introduction

Open surgical clipping has been generally considered the best treatment option for unruptured middle cerebral artery aneurysms (uMCAAs) (1). Some meta-analyses comparing coiling vs. microsurgical clipping in the treatment of uMCAAs have suggested that surgical clipping remains safe and more effective than endovascular coiling (2, 3). Moreover, the results of the randomized CURES trial (Collaborative UnRuptured Endovascular vs. Surgery) showed that open surgical management of uMCAAs resulted in better efficacy than endovascular management (4).

The difficult anatomical characteristics at this location have been attributed to the limitations of endovascular treatment of uMCAAs. However, new endovascular devices have been introduced to overcome the anatomical challenges that these aneurysms involve. Currently, this type of aneurysm is the target of these new devices relying on their, often, self-attributed and barely proven ability to succeed in complex anatomical sets. This is leading to a paradigm shift for treating uMCAAs with little space for actual, thorough, and sound comparison as it would be scientifically desired. Moreover, the bibliography reporting the virtues of these novel endovascular tools usually starts with the widely accepted statement “the ISAT demonstrated significant risk reduction after endovascular treatment compared with surgical clipping,” while they are actually analyzing techniques and situations completely different from the referenced study.

**Figure 1:**
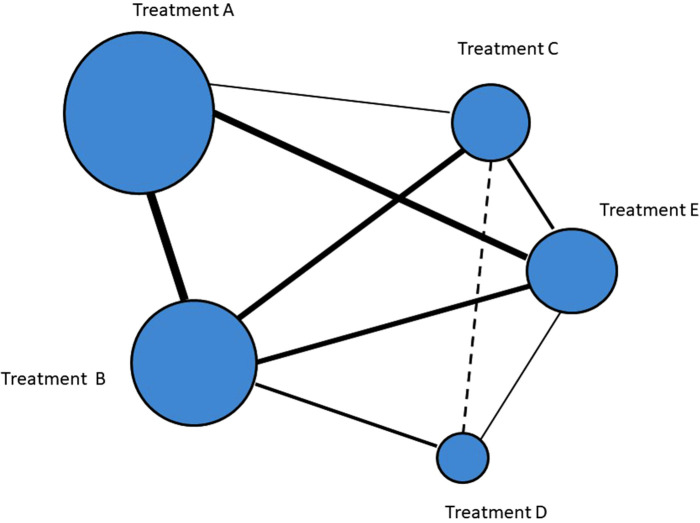
Graphical representation of a network meta-analysis: each ellipse (node) represents a treatment, with its size proportional to the number of cohorts in the node. Connections with solid lines between nodes represent direct comparative studies between treatments with the weight of the connecting lines representing the volume of direct comparative evidence. Connections with dashed lines represent indirect comparisons.

Network meta-analysis (NMA) is a statistical technique for comparing multiple treatments in a single analysis by combining direct and indirect evidence within a network of studies (Figure 1). Network meta-analysis may assist in assessing the comparative effectiveness of different treatments regularly used in clinical practice (5). Given that the treatment of uMAAs is currently being carried out through different techniques, this type of analysis could be the best in order to establish the pros and cons of the different types of management.

Due to the known lack of randomized controlled studies in our field enough to reach our purpose, we have carried out a systematic review of randomized and quasi-experimental studies to conduct a network meta-analysis of the different treatment methods currently used in uMCAAs.

## Methods

We developed a detailed protocol including objectives and plans for collecting and analyzing data. This article was prepared in accordance with the PRISMA-NMA guidelines (6). The study was designed, conducted, analyzed, and written independently from the industry and did not receive any other financial support to disclose.

### Search strategy

A systematic literature search of PubMed and Google Scholar was performed from January 2010 to December 2021, with no limits on languages. The keywords and free text searches used in combination (by using the Boolean operators OR and AND), were the following: “MCA aneurysm(s) “middle cerebral artery aneurysm(s), “unruptured”, “endovascular”, “microsurgery”, “clipping”, “coiling”, “flow diversion”, “flow diverter”, “WEB device”, “pCONus”, “stent assisted-coiling”, “balloon coiling”, “remodeling”, “contour device”, “PulseRyder device”, “comaneci device”. Duplicates articles were removed, and articles that had an irrelevant title and abstract, outcomes, and inadequate information were also rejected. The remaining publications were further assessed by reading the full text.

### Selection criteria

The inclusion criteria for the studies were the following: (a) quasi-experimental studies that include at least two arms; (b) studies with at least five cases per arm; (c) reporting the duration of follow-up and number of lost cases; (d) documenting in some way the rate of aneurysm occlusion during the follow-up and retreatments; (d) documenting post-op death or neurological complications; (e) reporting outcome with the modified Rankin scale (mRS), the Glasgow Outcome Scale (GOS), or the Extended Glasgow Outcome Scale (GOSE) during the follow-up.

The exclusion criteria for the studies were the following: (a) one-arm studies, (b) if the data of the subset of cases of uMCAAs were impossible to extract, and (c) the patients should be adults (>18 years)

### Quality analysis

Two authors (SC and SG-G) independently graded the quality of the studies using the Risk Of Bias In Non-randomised Studies of Interventions (ROBINS-I), a tool for assessing the risk of bias in nonrandomized studies of interventions (7). When the grading score given by the reviewers was in disagreement, RS acted as referee.

### Data extraction

Only uMCAAs were included. All patients with aneurysms found in the context of subarachnoid hemorrhage were excluded from the analysis.

### End points

– Angiographic success: complete or near-complete occlusion of the treated aneurysm at the end of the follow-up.
– Late outcome: We define bad results as patients who have died or who have neurological sequelae that force them to require help with their activities at the end of follow-up (mRS >4; GOS and GOSE ≤4).
– Retreatment: Need for repair of the treated aneurysm during follow-up.

### Data synthesis and analysis

The protocol included a standard pairwise meta-analysis for the included first-line therapies compared with at least two studies using Revman 5.2. The risk ratios (RRs) with 95% confidence intervals (CIs) were extracted employing the Cochran–Mantel–Haenszel test. In addition, Cochran's Q test and I2 statistic were determined to estimate the heterogeneity among the included studies. A fixed-effects model was considered when no inconsistency was found after the random-effect model test analysis.

After that, the protocol was programmed to analyze direct and indirect comparisons of the treatments through NMA using R-Studio. Dichotomous variables including complete or near-complete occlusion, adverse events, and the clinical outcome would be analyzed by calculating the RRs with their 95% CIs as the summary statistic. The rank plots based on the probabilities of the different interventions would be calculated.

## Results

### Study characteristics

**Figure 2:**
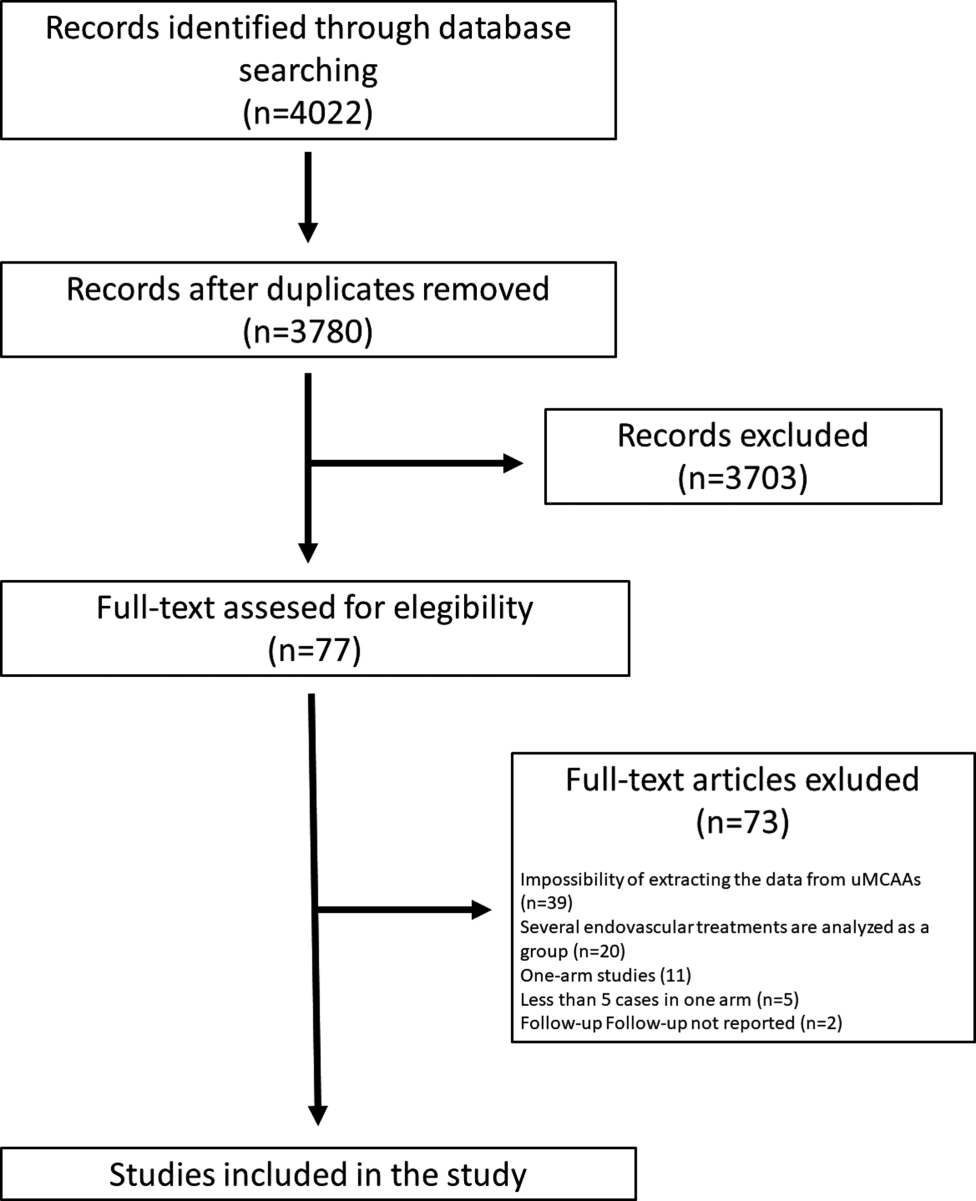
Flow diagram showing the flow of information through the different phases of the systematic review.

Only four quasi-experimental studies with 398 uMCAAs were included for analysis (Figure 2) (8–11). No new studies were found in the reference lists. All of them were unicenter studies. The four analyzable studies compared clipping vs. coiling. No other quasi-experimental study comparing other types of devices or techniques was found, so we could not conduct the network meta-analysis provided for in the protocol.

Using ROBINS-I, both reviewers rated all four articles as “moderate risk of bias studies.” This is defined as the following: “the studies are sound for a non-randomised study with regard to this domain but cannot be considered comparable to a well performed randomised trial”. In all studies, one or more authors were the physicians performing the treatment and, indeed, judging the outcome. The median year of publication was 2014 (range = 2011–2020). Cases were collected from 1999 to 2015.

**Figure 3:**
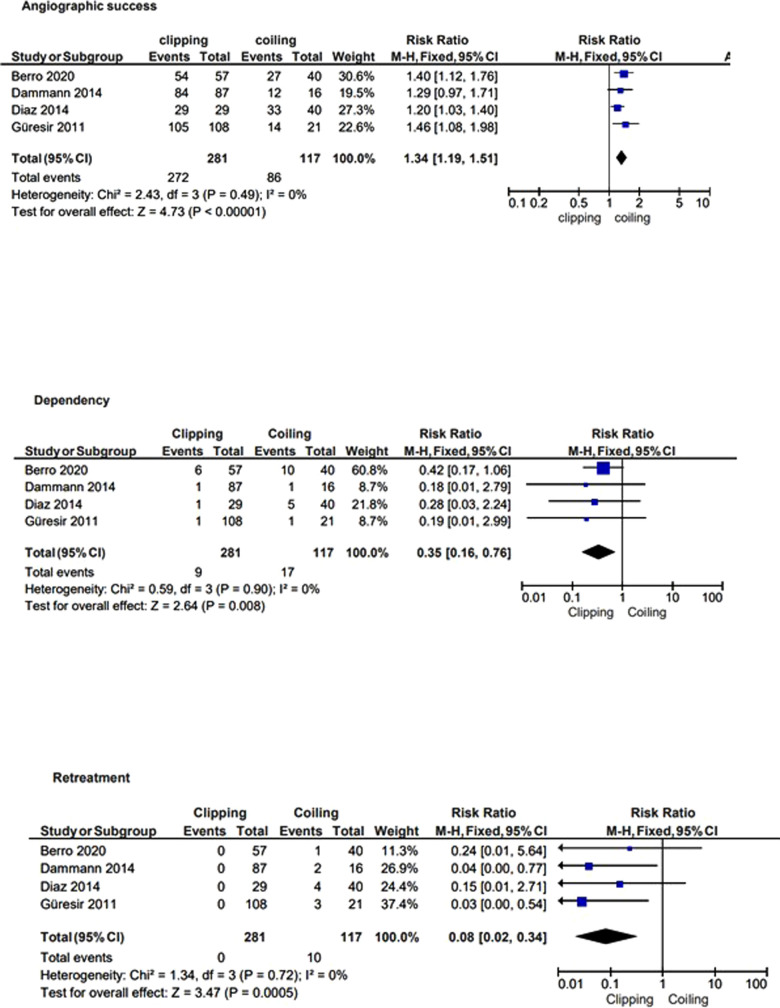
Forest plot showing RRs of clipping vs. coiling in terms of angiographic occlusion, final outcome, and retreatments.

The median angiographic follow-up was 11.5 months (range 6–48). Clipping was shown to be statistically significantly superior to coiling in terms of duration of aneurysmal occlusion [RR: 1.34 (95% CI: 1.19–1.51)]. The median clinical outcome follow-up was 12 months (range 6–12) with a long-term outcome better in the clipping arm [RR: 0.35 (95% CI: 0.16–0.76)]. Likewise, the need for retreatment was lower in the group treated by clipping [RR: 0.08 (95% CI: 0.02–0.34)]. Heterogeneity was low between the studies (Figure 3).

## Discussion

The results of our meta-analysis reinforce the data from previous authors that indicated the superiority of clipping treatment over coiling in uMCAAs (2, 3).

However, a new trend assumes that the advancement of newly designed devices can overcome this obstacle and, therefore, make endovascular therapy the first-line treatment (12). The results of our systematic review show that this trend may be supported by poor levels of evidence.

### Balloon remodeling technique

The first device widely employed to solve the problem of embolization of wide-neck aneurysms and the subsequent risk of coil protrusion was endoluminal balloons. Whether the use of balloon remodeling technique (BRT) carries a higher risk of complications has been controversial, with series indicating an increase in thromboembolic and hemorrhagic events and others not showing an increased risk (13, 14). In a recent multicenter study conducted in 16 French centers, data on 1,189 ruptured and unruptured aneurysms were collected (15). In this study, the usefulness of BRT vs. coiling alone could not be analyzed because BRT was heterogeneously used from one center to another (13.1%–94.4%, mean 45.8%), and in the centers with high use of balloon assisted coiling (BAC), a higher percentage of ruptured aneurysms were treated. In the study, immediate postoperative aneurysm occlusion was complete occlusion in 57.8%, neck remnant in 34.4%, and aneurysm remnant in 7.8%. Adequate occlusion (complete occlusion or neck remnant) was significantly more frequent in aneurysms with a size <10 mm, in aneurysms with a narrow neck, and in patients aged <70 years. In this scenario, it is important to highlight the recent publication of the uMCAAs subgroup of the randomized CURES study (4). Randomized data from this trial show that better efficacy may be obtained with surgical management of patients with uMMAAs. In this trial, simple coiling with/without balloon assistance was used in 92% of patients with the endovascular arm.

Comaneci embolization assist device (CEAD) is a compliant, adjustable mesh that provides temporary scaffolding during coiling of wide-necked intracranial aneurysms, preserving antegrade flow at the difference from classical BRT, but there is no study comparing CEAD vs. BRT.

### Stent-assisted coiling

Stent-assisted coiling (SAC) is one of the most used techniques for wide-neck aneurysms. Discordant data can be found in series regarding the increase or not of complications and the rate of angiographic occlusion (16, 17). One meta-analysis comparing clinical outcomes of aneurysms treated with SAC vs. BRT proposed that SAC achieved better complete occlusion rates of aneurysms 6 months or later after the procedure compared to BRT without higher risk of intraprocedural complications. Gory et al. reported their results of endovascular treatments of their subset of MCA aneurysms and found that SAC increases the risk of procedural complications.

With the intent to improve the capability of supporting the coils into the aneurysms, the Barrel vascular reconstruction device (BVRD) was developed. BVRD is a self-expandable, laser-cut stent that has a bulged center section to herniate over the aneurysmal ostium. Using a prospective, multicenter, observational post-marketing registry evaluating the use of the BVRD for treatment of wide-necked bifurcation aneurysms, Gory et al. found that BVRD resulted in ∼80% occlusion rates and ∼5% rates of neurological complications at 1 year after treatment (18). In another multicenter study conducted by Kabbasch et al. (19) with 21 intracranial aneurysms, 95% of them were occluded after a median of 282 days. However, as in the rest of the designed stents, there are no randomized or quasi-experimental studies comparing this device with other techniques used for treating of uMCAAs.

### Flow-diverter devices

Flow-diverter devices (FDDs) emerged as a new generation of endoluminal implants that were designed to treat aneurysms by reconstructing the diseased parent artery, creating initial great enthusiasm. However, great heterogeneity and publication biases were detected in the first series (20). Several years after the massive introduction of this device in clinical practice, the results of The Flow Diversion in the Treatment of Intracranial Aneurysm Trial (FIAT) were reported (21). That study pointed out that FDDs were not as safe and effective as had been hypothesized. Actually, the study should be halted because of safety concerns. When other nonrandomized studies have restricted the analysis to the use of FDDs in MCA aneurysms, the results are even more disappointing. A meta-analysis reported by Cagnazzo et al. (22), focused on the use of FDDs in MCA aneurysms, showed that the overall rate of complete/near-complete occlusion during follow-up was 78.7% (95% CI: 67.8%–89.7%) with a 12-month median duration, and the rate of treatment-related complications was 20.7% (95% CI: 14%–27.5%) of which approximately 10% was permanent. More recently, Diestro et al. (23) published an international cohort study of 54 MCA bifurcation aneurysms compared to the published series on open surgical treatment; FDDs have inferior outcomes and are associated with a higher rate of complications. In this report, 16.7% (9/54) of the patients suffered a thromboembolic complication, and from the 45 aneurysms with available follow-up data, 20% did not have adequate occlusion with a median follow-up time of 12 months.

### Endoaneurysmatic devices

The enthusiasm about intrasaccular flow disrupting devices is great at this moment (24).

Woven Endo-Bridge (WEB) is a Nitinol-based braided wire designed to be introduced into intracranial aneurysms, where it spans the aneurysmal neck to disrupt inflow and reduce intrasaccular flow. The novel and promising aspect attributed to WEB was that, due to the device being positioned into the aneurysm lumen, it does not require dual antiplatelet agents, which would be especially useful in ruptured aneurysms. The largest study in terms of participant centers published so far has been the CLARYS study (25). In this multicenter study, 60 patients with 60 ruptured bifurcation aneurysms to be treated with the WEB were included, and the interim results appeared to be promising. Essibayi et al. (26) and Harker et al. (27) have published systematic reviews and meta-analyses about the use of WEB for ruptured aneurysms. Both of them showed promising results but were focused on ruptured aneurysms, and the risk of bias may be high because they are based on single-arm clinical experiences without a control group. At this moment, a trial is ongoing [The RISE Trial (28)], which is a multicenter, randomized trial including ruptured and unruptured aneurysms.

Medina embolization device (MED) is a hybrid embolization device that combines the properties of a conventional coil with those of an intrasaccular flow disrupter. The results of MED have been only published by groups explaining their early experiences of groups, including one-arm series of few patients, and with heterogeneous results (29–32).

Contour device is the last introduction in this so promising group of intrasaccular flow disrupting devices. Recently, the results of The Safety and Effectiveness of the Contour Neurovascular System (Contour) for the Treatment of Bifurcation Aneurysms (The CERUS Study), have been published (33). This study concludes that the Contour seems to be both safe and effective for treating intracranial bifurcation aneurysms. It is noteworthy to mention that the conclusions of this study are based on the results of 30 aneurysms and that the rate of obliteration at 6 months was 69%.

### Neck supporting devices

PulseRider Device (PRD) is a permanent Nitinol (nickel-titanium) self-expanding stent implant for treating wide-necked aneurysms located at or near branching areas. A recent meta-analysis has been published analyzing the data on PRD for treating wide-necked intracranial aneurysms (34). Although the study showed that PRD reached a 90% adequate occlusion rate that increases up to 91% in the sixth month with a 5% complication rate, the results were obtained from 157 subjects from six one-arm series. Additionally, no analysis of heterogeneity or bias is performed.

pCONus is a device with four petals and is implanted inside the aneurysm at its neck, whereas the shaft is anchored in the parent vessel. Krupa et al. performed a meta-analysis (35). They found eight one-arm studies for analysis, and the effectiveness and safety of the device were considered to be “moderate.” More recently, the results of the pToWin study, a prospective, single-arm, multicenter study conducted to analyze the safety and efficacy of the pCONus, have been published (36). In terms of safety, the morbidity-mortality rate was lower than that reported in the meta-analysis of Krupa et al., but only 75.0% and 65.6% showed adequate occlusion at 3–6 and 7–12 months, respectively.

### Limitations

The main limitation of our study, of course, is that we have not been able to conduct the network meta-analysis that we had proposed. The absence of randomized or quasi-experimental studies has prevented the performance of such an analysis. This problem has happened before. In a study conducted by Graziano et al., aimed at evaluating the efficacy and safety of different treatments in aneurysms of the vertebrobasilar region, the authors failed to realize the intended meta-analysis (37). But, on the other hand, the fact that it is impossible to carry out a network analysis on the treatment in cases of uMCAAs due to the absence of comparative studies when in practice procedures are being performed using new different devices should lead us to rethink the direction taken. The neurovascular community should avoid feeling overexcited immediately after the appearance of new theoretically perfect solutions. Using new devices faster than the scientific verification would allow might be considered an inappropriate practice. There is an urgent need for comparative studies on the treatment of uMCAAs.

## Conclusions

Surgical clipping showed better long-term results than endovascular coiling in the treatment of uMCAAs. After carrying out this systematic review, and the impossibility of conducting NMA due to the absence of randomized or quasi-experimental studies, we must call for scientific prudence and the need to initiate trials in order to increase our knowledge in the treatment of uMCAAs

## Data Availability

The original contributions presented in the study are included in the article/Supplementary Material, further inquiries can be directed to the corresponding authors.
